# Cannabidiol Modulates Alterations in PFC microRNAs in a Rat Model of Depression

**DOI:** 10.3390/ijms24032052

**Published:** 2023-01-20

**Authors:** Uri Bright, Irit Akirav

**Affiliations:** 1 Department of Psychology, School of Psychological Sciences, University of Haifa, Haifa 3498838, Israel; 2 The Integrated Brain and Behavior Research Center (IBBRC), University of Haifa, Haifa 3498838, Israel

**Keywords:** depression, cannabidiol, microRNAs, CB1 receptor, 5-HT1a receptor, prefrontal cortex

## Abstract

Cannabidiol (CBD) is a potential antidepressant agent. We examined the association between the antidepressant effects of CBD and alterations in brain microRNAs in the unpredictable chronic mild stress (UCMS) model for depression. UCMS male rats were injected with vehicle or CBD (10 mg/kg) and tested for immobility time in the forced swim test. Alterations in miRNAs (miR16, miR124, miR135a) and genes that encode for the 5HT1a receptor, the serotonergic transporter SERT, β-catenin, and CB1 were examined. UCMS increased immobility time in a forced swim test (i.e., depressive-like behavior) and altered the expression of miRNAs and mRNA in the ventromedial prefrontal cortex (vmPFC), raphe nucleus, and nucleus accumbens. Importantly, CBD restored UCMS-induced upregulation in miR-16 and miR-135 in the vmPFC as well as the increase in immobility time. CBD also restored the UCMS-induced decrease in htr1a, the gene that encodes for the serotonergic 5HT1a receptor; using a pharmacological approach, we found that the 5HT1a receptor antagonist WAY100135 blocked the antidepressant-like effect of CBD on immobility time. Our findings suggest that the antidepressant effects of CBD in a rat model for depression are associated with alterations in miR-16 and miR-135 in the vmPFC and are mediated by the 5HT1a receptor.

## 1. Introduction

Depression is one of the most common psychiatric disorders worldwide [1]. Antidepressants are the current recommended standard of treatment for depression; however, their effectiveness is only slightly efficacious compared to placebos [2]. 

There has been growing evidence that cannabidiol (CBD) may have therapeutic effects on depressive symptoms. Given its safety profile, CBD is a promising treatment for mood disorders; however, the exact molecular mechanisms underlying its potential antidepressant effects are largely unknown. Pre-clinical studies demonstrated the antidepressant effects of CBD, expressed as lower immobility time in the forced swimming test (FST) (i.e., less despair) [3,4,5] and higher saccharine consumption in the saccharine preference test (i.e., lower anhedonia) [3,6]. In mice that underwent olfactory bulbectomy (OBX), a model for depression, CBD improved depressive-like symptoms and elevated serotonin and glutamate levels in the prefrontal cortex (PFC) [7]. Human studies suggest that CBD has ameliorating effects in several disorders, which are in high comorbidity with depression, such as insomnia, borderline personality disorder, and social anxiety [8,9]. 

CBD has a very low affinity for both cannabinoid CB1 and CB2 receptors [10], but it modulates endocannabinoid function through its ability to inhibit the hydrolysis of anandamide and to act as a transient receptor potential vanilloid 1 agonist. Another major mediating pathway for CBD-anti-depressive effects may be through the serotonergic 5-HT1a receptor [4,11,12,13]. CBD administered into the medial PFC (mPFC) was found to induce antidepressant-like effects in the FST through indirect activation of CB1r and 5-HT1a [13]. Repeated administration of CBD was found to prevent long-lasting anxiogenic effects promoted by a single predator exposure; pretreatment with the 5-HT1a antagonist WAY100635 attenuated the CBD effects, suggesting the involvement of 5-HT1a in the mediation of those effects [11].

CBD activates the extracellular signal-regulated kinase (ERK) pathway through the 5-HT1a receptor [14], resulting in β-catenin accumulation in the cytosol and therefore in the cell nucleus [15]. β-catenin is a multi-functional protein that plays an important role in the mature central nervous system; its dysfunction has been implicated in several neuropsychiatric disorders, including depression [16]. We have recently found, that downregulating β-catenin levels in the nucleus accumbens (NAc), blocked the therapeutic-like effects of the fatty acid amide hydrolase (FAAH) inhibitor URB597 on anxiety- and depression-like behaviors in rats exposed to a rat model of post-traumatic stress disorder (PTSD) [17]. It has been shown that β-catenin is a critical regulator in the development of behavioral resilience, activating a network that includes downstream microRNAs (miRNAs, miRs) [18]. miRNAs are small non-coding RNA molecules comprising 19–25 nucleotides. miRNAs are implicated in a range of psychiatric disorders including anxiety and depression [19,20,21,22,23].

Different miRNAs target different mRNAs, leading to various effects, and behavioral phenotypes such as resilience to panic, anxiety, stress, etc. [24,25]. Several miRNAs have been associated with resilience to stress and depression; amongst them are miR-16, miR-124, and miR-135 [18,26,27,28,29,30].

MiR-16 was found to be less abundant in the cerebrospinal fluid of patients with major depression [31] and lower levels of miR-16 in the NAc were associated with susceptibility to stress in mice [18]. Moreover, MiR-16 modulates the expression of the serotonin transporter (SERT), a major target for SSRIs [31,32]. In raphe cells, elevated levels of miR-16 induced a decrease in the expression of SERT [31,32]. We have recently found that in adult males and females, exposed to early life stress (ELS), the FAAH inhibitor URB597 restored an ELS-induced decrease in mPFC miR-135a in females and miR-16 in males and the associated depressive-like phenotype in both sexes [33].

In the PFC, early adolescent stress downregulated miR-135a expression [34], while upregulating in the hippocampus [26,34], suggesting a brain-region-dependent effect. MiR-135a levels were significantly lower in the blood and brain of depressed human patients. MiR-135- knockdown also prompted an increase in 5-HT1a and SERT levels in the raphe nucleus [27]. A similar effect of miR-135a downregulation on 5-HT1a overexpression was seen in the PFC [34]. 

MiR-124, like miR-135, is a brain-specific miRNA [35]. A decrease in hippocampal miR-124 levels was correlated with depressive-like behaviors in mice that underwent chronic ultra-mild stress (CUMS), while overexpression of miR-124 enhanced behavioral resilience. MiR-124 targets the GSK3β-coding mRNA, as miR-124 overexpression induced GSK3β downregulation [28]. GSK3β is an enzyme that plays a role in β-catenin regulation [15,36,37,38], and its inhibition elevates cytosolic β-catenin, which promotes resilience to depression.

The aim of the present study was to examine in the unpredictable chronic mild stress (UCMS) model for depression, whether the antidepressant properties of CBD are associated with alterations in miRNAs implicated in depression (miR-16, miR-124, and miR-135) and with important target genes of these miRNAs and CBD. We examined the expression of genes that encode for the serotonergic 5HT1a receptor and SERT, and the expression of genes that encode for β-catenin and CB1. This was examined in the ventromedial PFC (vmPFC), NAc, and raphe nucleus, brain regions highly associated with the etiology of depression [39,40,41].

## 2. Results

### 2.1. The Effects of Chronic CBD Administration during UCMS on Behavior

We examined the effects of chronic CBD administration (10 mg/kg, i.p.) during the last 3 weeks of a 6-week UCMS model on immobility in the FST and on motoric and anxiety-like behavior in the open field test (OFT). All analyses were conducted using two-way ANOVA [stress × drug (2 × 2)].

**Figure 1:**
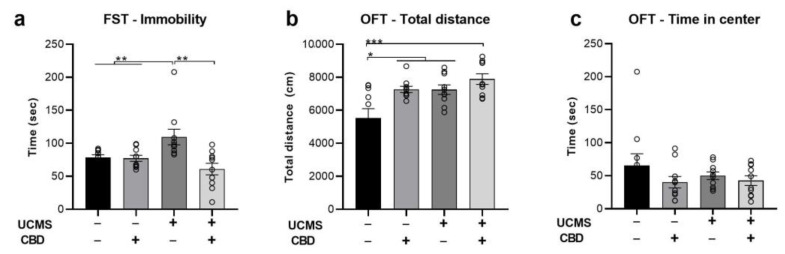
The effects of CBD treatment on behavior in rats exposed to UCMS. (**a**) FST: UCMS rats treated with a vehicle spent more time immobile than the No UCMS groups and UCMS rats treated with CBD. (**b**) OFT total distance: No UCMS rats treated with the vehicle covered less distance compared to all groups. (**c**) OFT time in the center: no differences between groups were observed. FST—forced swim test; OFT—open field test; UCMS: unpredictable chronic mild stress; CBD: cannabidiol. *, *p* < 0.05, **, *p* < 0.01, ***, *p* < 0.001.

In the FST (Figure 1a), we found a significant effect of drug (F(1,39) = 9.801, *p* < 0.01) and stress × drug interaction (F(1,39) = 8.732, *p* < 0.01) with no effect of stress (F(1,39) = 0.874, ns), suggesting that CBD restored UCMS-induced increase in immobility.

In the OFT, we found a significant effect of drug (F(1,39) = 10.586, *p* < 0.01) and stress (F(1,39) = 10.34, *p* < 0.01) on locomotion (Figure 1b), with no effect of stress x drug interaction (F(1,39) = 2.236, ns), suggesting that CBD and UCMS increased locomotion behavior compared with the No UCMS vehicle group. Furthermore, we found no effect of stress (F(1,39) = 0.355, ns), drug (F(1,39) = 2.219, ns), or stress × drug interaction (F(1,39) = 0.653, ns) on the time spent in the center of the arena during the first 5 min of the test (Figure 1c).

### 2.2. The Effects of Chronic CBD Administration during UCMS on miRNA Expression

Following the behavioral tests, we assessed the expression of miR-16, miR-124, and miR-135 in the vmPFC, NAc, and raphe nucleus. All analyses were conducted using two-way ANOVA [stress × drug (2 × 2)].

#### 2.2.1. miR-16

**Figure 2:**
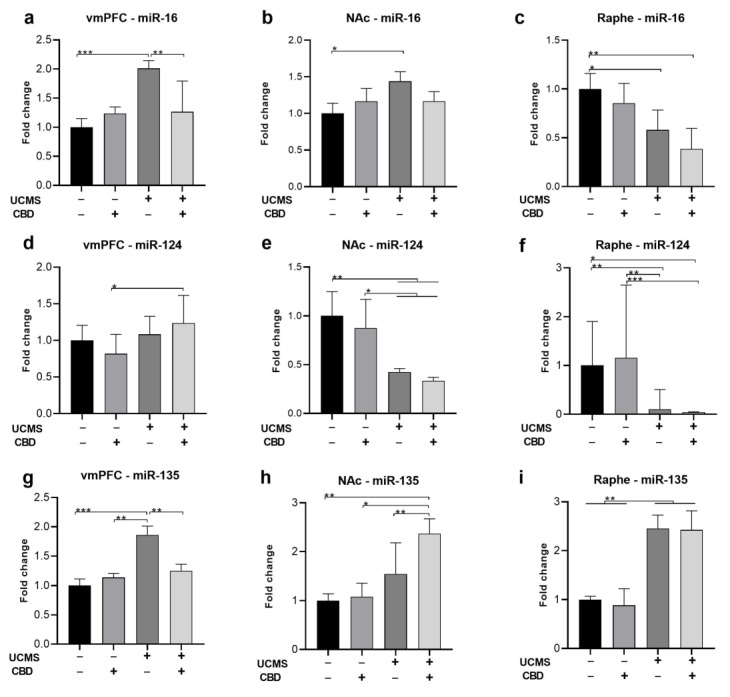
The effects of CBD treatment on miRNA expression in the vmPFC, NAc, and raphe nucleus in rats exposed to UCMS. miR-16. (**a**) vmPFC: UCMS rats treated with a vehicle demonstrated miR-16 upregulation compared to all groups. (**b**) NAc: UCMS rats treated with a vehicle demonstrated miR-16 upregulation compared to No UCMS rats treated with a vehicle. (**c**) Raphe: UCMS (vehicle, CBD) downregulated miR-16 compared to UCMS rats treated with a vehicle. miR-124: (**d**) vmPFC: UCMS rats treated with CBD demonstrated miR-124 upregulation compared to No UCMS rats treated with CBD. (**e**) NAc: UCMS downregulated miR-124 compared to No UCMS. (**f**) Raphe: UCMS downregulated miR-124 compared to No UCMS. miR-135: (**g**) vmPFC: UCMS rats treated with a vehicle demonstrated miR-135 upregulation compared to all groups. (**h**) NAc: UCMS rats treated with CBD demonstrated miR-135 upregulation compared to all groups. (**i**) Raphe: UCMS upregulated miR-135 compared to No UCMS. vmPFC: ventromedial prefrontal cortex; NAc: nucleus accumbens; miR: microRNA; UCMS: unpredictable chronic mild stress; CBD: cannabidiol. *, *p* < 0.05, **, *p* < 0.01, ***, *p* < 0.001.

In the **vmPFC** (Figure 2a), we found a significant effect of stress (F(1,35) = 15.648, *p* < 0.001), drug (F(1,35) = 4.276, *p* < 0.05), and stress x drug interaction (F(1,35) = 10.682, *p* < 0.01) on the expression of miR-16, suggesting that CBD restored UCMS-induced upregulation of miR-16. 

In the **NAc** (Figure 2b), a significant effect of stress (F(1,34) = 5.454, *p* < 0.05), with no effect of drug (F(1,34) = 0.001, ns) or stress x drug interaction (F(1,34) = 2.434, ns) was observed, suggesting that UCMS upregulated miR-16 compared to the controls, an effect that was not observed in CBD-treated rats.

In the **raphe** (Figure 2c), we found a significant effect of stress (F(1,29) = 15.141, *p* < 0.001), with no effect of drug (F(1,29) = 2.862, ns) or stress × drug interaction (F(1,29) = 0.18, ns), suggesting that UCMS downregulated miR-16, with no effect for CBD.

#### 2.2.2. miR-124

In the **vmPFC** (Figure 2d), we found a significant effect of stress (F(1,35) = 7.668, *p* < 0.01), with no effect of drug (F(1,35) = 0.155, ns), or stress x drug interaction (F(1,35) = 3.507, ns) on the expression of miR-124, suggesting that UCMS upregulated miR-124 in CBD-treated rats.

In the **NAc** (Figure 2e), and in the **raphe** (Figure 2f), a significant effect of stress (NAc: F(1,36) = 25.341, *p* < 0.001; raphe: F(1,37) = 30.532, *p* < 0.001) was observed, with no effect of drug (NAc: F(1,36) = 1.063, ns; raphe: F(1,37) = 0.527, ns) or stress × drug interaction (NAc: F(1,36) = 0.087, ns; raphe: F(1,37) = 1.03, ns), suggesting that UCMS downregulated miR-124, with no effect for CBD. 

#### 2.2.3. miR-135

In the **vmPFC** (Figure 2g), we found a significant effect of stress (F(1,33) = 16.546, *p* < 0.001), drug (F(1,33) = 4.745, *p* < 0.05), and stress × drug interaction (F(1,33) = 8.748, *p* < 0.01) on the expression of miR-135, suggesting that CBD restored UCMS-induced upregulation of miR-135.

In the **NAc** (Figure 2h), we found a significant effect of stress (F(1,34) = 6.996, *p* < 0.05) and drug (F(1,34) = 12.672, *p* < 0.01) with no effect of stress x drug interaction (F(1,34) = 2.726, ns), suggesting that CBD upregulated miR-135 only in UCMS rats. 

In the **raphe** (Figure 2i), we found a significant effect of stress (F(1,27) = 19.081, *p* < 0.001), with no effect of drug (F(1,27) = 0.28, ns) or stress × drug interaction (F(1,27) = 0.187, ns), suggesting that UCMS upregulated miR-135, with no effect for CBD.

Pearson bivariate correlations tests (Table 1) were conducted between miRNA expression in the different brain regions and the behavioral measures to explore the association between the depressive-like behavior of the rats and their miR expression. For immobility, the most robust effect was observed with vmPFC miR-135 levels (r = 0.428, *p* < 0.05), suggesting that increased immobility was associated with vmPFC miR-135 upregulation. A negative correlation was observed with NAc miR-135 levels (r = −0.339, *p* < 0.05).

For total distance in the OFT, significant correlations were observed with NAc miR-124 (r = 0.455, *p* < 0.01), raphe miR-135 (r = 0.474, *p* < 0.05), and raphe miR-16 (r = −0.473, *p* < 0.01) levels. These suggest that increased locomotion behavior was associated with increased NAc miR-124 and raphe miR-135 and decreased raphe miR-16.

### 2.3. The Effects of Chronic CBD Administration during UCMS on Possible Target Genes

Previous findings suggested that the effects of CBD on depressive-like behavior may be mediated via serotonergic mechanisms and CB1r activation [4,11-13]. We have recently shown that the stress-preventing effects of FAAH inhibition are mediated by β-catenin [17]. Hence, we next examined alterations in the expression of several target genes in UCMS rats treated with CBD. 

We examined the expression of htr1a and slc6a4 genes that encode for the serotonergic 5HT1a receptor and SERT, respectively, and the expression of ctnnb1 and cnr1, the genes that encode for β-catenin and CB1, respectively. All analyses were conducted using two-way ANOVA [stress × drug (2 × 2)].

#### 2.3.1. htr1a

**Figure 3:**
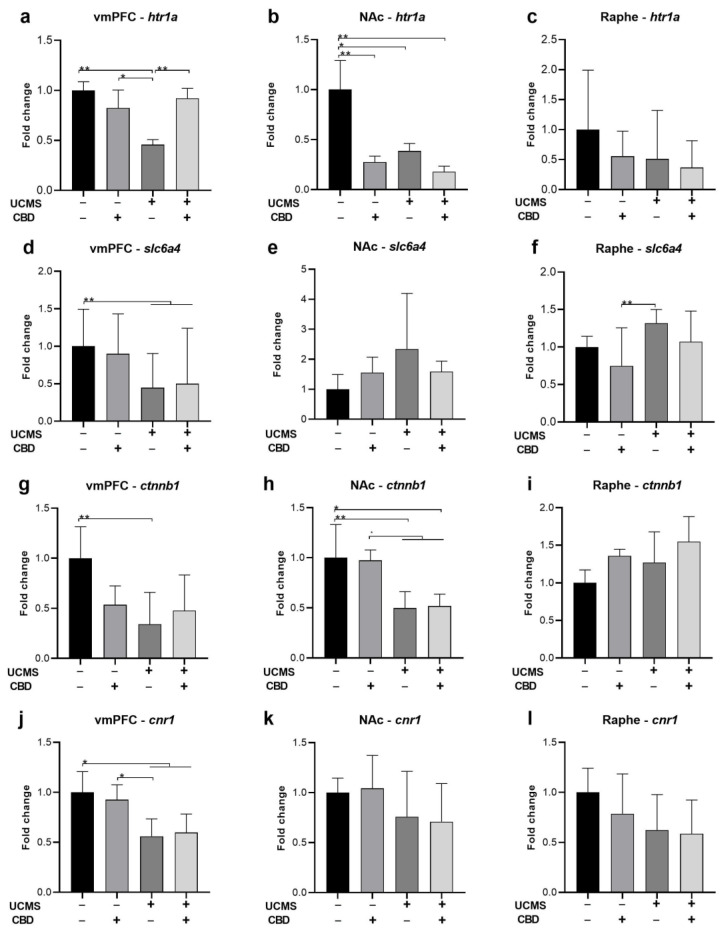
The effects of CBD treatment on serotonergic, β-catenin, and CB1 mRNA expression in the vmPFC, NAc, and raphe nucleus in rats exposed to UCMS. htr1a: (**a**) vmPFC: UCMS-vehicle downregulated htr1a levels compared to all groups. (**b**) NAc: UCMS rats treated with a vehicle or CBD and No UCMS rats treated with CBD demonstrated htr1a downregulation compared to No UCMS rats treated with a vehicle. (**c**) Raphe: no differences were observed between the groups. slc6a4: (**d**) vmPFC: UCMS rats treated with a vehicle or CBD demonstrated downregulation compared to No UCMS rats treated with a vehicle. (**e**) NAc: no differences were observed between the groups. (**f**) Raphe: UCMS-vehicle rats demonstrated upregulation of slc6a4 compared to No UCMS-CBD rats. Ctnnb1: (**g**) vmPFC: UCMS-vehicle rats demonstrated Ctnnb1 downregulation compared to No UCMS-vehicle rats. (**h**) NAc: UCMS downregulated Ctnnb1 compared to No UCMS rats treated with a vehicle or CBD. (**i**) Raphe: no differences were observed between the groups. Cnr1: (**j**) vmPFC: UCMS rats that were treated with a vehicle or CBD demonstrated Cnr1 downregulation compared to No UCMS rats treated with a vehicle or CBD. (**k**) NAc: no differences were observed between the groups. (**l**) Raphe: no differences were observed between the groups. vmPFC: ventromedial prefrontal cortex; NAc: nucleus accumbens; UCMS: unpredictable chronic mild stress; CBD: cannabidiol. *, *p* < 0.05, **, *p* < 0.01.

In the **vmPFC** (Figure 3a)**,** we found a significant effect of stress (F(1,35) = 5.586, *p* < 0.05) and stress x drug interaction (F(1,35) = 9.861, *p* < 0.01) but not for drug (F(1,35) = 3.169, ns), suggesting that CBD restored UCMS-induced downregulation of htr1a. 

In the **NAc** (Figure 3b), we found a significant effect of stress (F(1,26) = 6.916, *p* < 0.05), drug (F(1,26) = 10.584, *p* < 0.01) and stress x drug interaction (F(1,26) = 4.386, *p* < 0.05), suggesting that UCMS and CBD downregulated htr1a. 

In the **raphe** (Figure 3c), we found no effect of stress (F(1,29) = 1.158, ns), drug (F(1,29) = 0.684, ns), or stress × drug interaction (F(1,29) = 0.305, ns), suggesting no effect for UCMS or CBD on htr1a.

#### 2.3.2. slc6a4

In the **vmPFC** (Figure 3d), we found a significant effect of stress (F(1,27) = 14.184, *p* < 0.01) but not of drug (F(1,27) = 0.49, ns)), or stress x drug interaction (F(1,27) = 0.438, ns), suggesting that UCMS downregulated slc6a4, with no effect for CBD.

In the **NAc** (Figure 3e), we found no effect of stress (F(1,25) = 0.259, ns), drug (F(1,25) = 0.319, ns), or stress × drug interaction (F(1,25) = 1.253, ns), suggesting no effect for UCMS or CBD on slc6a4.

In the **raphe** (Figure 3f), we found a significant effect of stress (F(1,27) = 5.272, *p* < 0.05), drug (F(1,27) = 6.904, *p* < 0.05) but not for stress x drug interaction (F(1,27) = 0.303, ns), suggesting that UCMS rats treated with vehicle showed upregulation compared to No UCMS-CBD rats.

#### 2.3.3. ctnnb1

In the **vmPFC** (Figure 3g), we found a significant effect of stress (F(1,35) = 7.864, *p* < 0.01) and stress × drug interaction (F(1,35) = 5.071, *p* < 0.05), but not for drug (F(1,35) = 0.453, ns) on the expression of ctnnb1, suggesting that in UCMS-vehicle, but not in UCMS-CBD rats, ctnnb1 was downregulated, an effect that was not observed in CBD-treated rats.

In the **NAc** (Figure 3h), a significant effect of stress (F(1,34) = 21.045, *p* < 0.001) was observed, but not of drug (F(1,34) = 0.001, ns) or stress × drug interaction (F(1,34) = 0.051, ns), suggesting that UCMS downregulated ctnnb1, with no effect for CBD.

In the **raphe** (Figure 3i), we found no effect of stress (F(1,33) = 0.157, ns), drug (F(1,33) = 0.496, ns), or stress × drug interaction (F(1,33) = 0.037, ns), suggesting no effect for UCMS or CBD on ctnnb1.

#### 2.3.4. cnr1

In the **vmPFC** (Figure 3j), we found a significant effect of stress (F(1,35) = 16.429, *p* < 0.001) but not for drug (F(1,35) = 0.002, ns) or stress × drug interaction (F(1,35) = 0.314, ns), suggesting that UCMS downregulated cnr1. In the **NAc** (Figure 3k) and **the raphe** (Figure 3l), we found no effect of stress (NAc: F(1,32) = 1.736, ns; raphe: F(1,30) = 2.111, ns), drug (NAc: F(1,32) = 0.003, ns; raphe: F(1,30) = 0.32, ns) or stress x drug interaction (NAc: F(1,32) = 0.048, ns; raphe: F(1,30) = 0.116, ns), suggesting no effect for UCMS or CBD on cnr1.

Pearson bivariate correlations tests were conducted between miRNA expression and genes in the various brain regions.

In the vmPFC (Table 2), the most robust correlations were observed between miR-16 and hrt1a (r = 0.591, *p* < 0.001) and slc6a4 (r = 0.432, *p* < 0.05), and between miR-135 and hrt1a (r = 0.478, *p* < 0.01), suggesting that levels of the 5HT1a and SERT genes were associated with these microRNAs.

In the NAc (Table 3), the most robust correlations were observed between miR-16 and ctnnb1 (r = 0.479, *p* < 0.01) and between miR-124 and ctnnb1 (r = 0.463, *p* < 0.01), suggesting that levels of these microRNAs were associated with the β-catenin gene.

In the raphe nucleus (Table 4), the most robust correlations were observed between miR-124 and slc6a4 (r = 0.408, *p* < 0.05) suggesting that levels of this microRNA were associated with SERT gene.

### 2.4. Does the 5-HT1a Antagonist WAY100635 Block the Effects of Chronic CBD Administration during UCMS on Behavior

As CBD was found to restore UCMS-induced downregulation of the htr1a gene that encodes for the serotonergic 5HT1a receptor, we pharmacologically examined whether the effects of CBD on behavior are mediated by the activation of the 5HT1a receptor. To that end, in a different set of animals, we assessed whether the antidepressant effects of CBD (see Figure 1) are mediated by the serotonergic 5HT1a receptor by administering a 5HT1a receptor antagonist. We administered the 5HT1a-antagonist WAY100635 (0.1 mg/kg) along with CBD every day during the last 3 weeks of UCMS. Other groups were injected with CBD or WAY or vehicle for comparison. All analyses were conducted using two-way ANOVA [stress × drug (2 × 2)].

**Figure 4:**
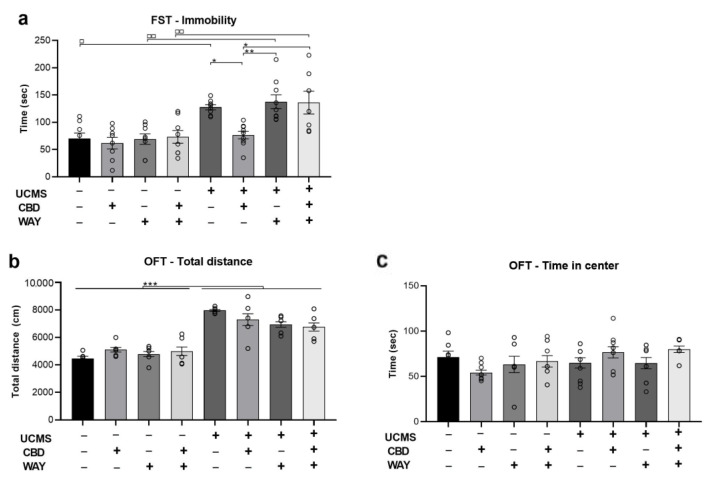
The effects of the 5-HT1a antagonist WAY100635 on behavior in rats exposed to UCMS and treated with CBD. (**a**) FST: UCMS rats that were treated with CBD, spent less time immobile than UCMS rats that were treated with vehicle, WAY, or CBD + WAY. Furthermore, UCMS rats treated with vehicle, WAY, or CBD + WAY, showed increased immobility compared to No UCMS rats injected with vehicle, WAY, or CBD + WAY, respectively. (**b**) OFT: UCMS rats traveled more than No UCMS rats. (**c**) OFT time in the center: no differences between groups were observed. FST—forced swim test; OFT—open field test; UCMS: unpredictable chronic mild stress; CBD: cannabidiol; WAY: WAY100635, *, *p* < 0.05; **, *p* < 0.01; ***, *p* < 0.001; compared to UCMS-CBD group ◻ *, *p* < 0.05, ◻◻ **, *p* < 0.01, ◻◻◻ ***, *p* < 0.001 white aquares indicate statistical significance in UCMS vs. NO UCMS groups.

In the FST (Figure 4a), we found a significant effect of stress (F(1,79) = 40.143, *p* < 0.001) and drug (F(1,79) = 4.506, *p* < 0.01) with no effect of stress x drug interaction (F(1,79) = 2.409, ns), suggesting that CBD restored UCMS-induced immobility, an effect that was blocked by the antagonist WAY. UCMS rats injected with vehicle, WAY, or WAY + CBD demonstrated increased immobility compared to No UCMS rats that were injected with vehicle (*p* < 0.05), WAY (*p* < 0.01), or WAY + CBD (*p* < 0.01), respectively.

In the OFT, we found a significant effect of stress (F(1,79) = 185.137, *p* < 0.001) and stress x drug interaction (F(1,79) = 4.602, *p* < 0.01) on locomotion (Figure 4b) with no effect of drug (F(1,79) = 1.227, ns), suggesting that UCMS increased locomotion, with no effect for CBD. Furthermore, we found no effect of stress (F(1,79) = 3.197, ns), drug (F(1,79) = 0.928, ns), and stress × drug interaction (F(1,79) = 2.210, ns) on the time spent in the center of the arena in the first 5 min of the test (Figure 4c).

## 3. Discussion

In this study, we show for the first time that CBD can restore UCMS-induced upregulation of miR-16 and miR-135 in the vmPFC as well as the associated despair-like behavior. UCMS also downregulated the 5-HT1a gene htr1a in the vmPFC; using a pharmacological approach with the 5-HT1a receptor antagonist WAY, we found that the antidepressant-like effects of CBD are mediated by the 5HT1a receptor. 

The antidepressant effects of CBD on despair-like behavior in the FST corroborates with previous findings [3,4,5,42,43,44]. In the open field, CBD did not restore the UCMS-induced increase in locomotion activity and UCMS had no significant effect on anxiety-like behavior measured as time spent in the center of the open field.

The expression of miR-16, miR-124, and miR-135 was significantly affected by UCMS, corroborating with previous studies demonstrating that these miRNAs are correlated with anxiety- and depressive-like phenotypes and are significantly downregulated or upregulated following stress exposure, depending upon the brain region studied and the type of stressor [18,23,26,27,28,29,45,46]. Specifically, we found that UCMS decreased the expression of miR-124 in the NAc and raphe and increased miR-16 in the NAc and the raphe and miR-135 in the raphe. However, these effects were not normalized by CBD treatment. Similarly, UCMS affected the target genes, decreasing slc6a4 and cnr1 expression in the vmPFC (genes coding SERT and CB1), and decreasing ctnnb1 (β-catenin) in the PFC and NAc, with no effect for CBD treatment. 

### 3.1. Alterations in miRNAs

#### 3.1.1. miR-16

In general, higher serum levels of miR-16 are associated with a resilient phenotype to stress [47], while patients with major depression exhibit lower CSF expression of the same microRNA [30,48]. Nevertheless, these findings are rarely associated with specific brain regions. In mice, social defeat stress was associated with susceptibility to stress and lower accumbal miR-16 [18], while in rats, ELS-induced stress was associated with lower miR-16 in the vmPFC [32]. 

It has been shown that maternally deprived rats, but not rats exposed to chronic unpredictable stress, showed higher hippocampal miR-16 expression than control rats [26]. Taken together these findings suggest that different stressors differentially affect the expression of miRs in a brain-region-dependent manner. 

#### 3.1.2. miR-124

In the vmPFC, UCMS rats that were treated with CBD had higher levels of miR-124 only compared to control rats who were treated with CBD. UCMS decreased miR-124 in the NAc and in the raphe nucleus with no effect of CBD treatment, suggesting that its antidepressant effects are mediated by a different mechanism.

Previous studies showed that rats that were treated chronically with corticosterone as a model of depression presented higher miR-124 levels in the PFC [49], and vmPFC suppression of miR-124 via lentiviral vector decreased depressive symptoms [50]. Other studies have shown a decrease in miR-124 in the hippocampus following UCMS and that using an agomir to increase miR-124 had an antidepressant-like effect [51]. Similarly, addictive behavior to cocaine was associated with lower NAc levels of this microRNA [52,53,54], thus indicating that miR-124 may play an important role in the reward system. However, in another study, UCMS increased hippocampal miR-124 expression, and downregulation of miR-124 using an antagomir decreased depressive-like behavior [55]. 

#### 3.1.3. miR-135

Previous findings showed decreased miR-135 in the PFC and raphe of mice exposed to chronic stress [27,33] and decreased PFC miR-135 in rats exposed to early life stress [32,33]. Downregulation of raphe miR-135 was observed following exposure to the chronic social defeat stress model in mice [27]. 

### 3.2. Alterations in Serotonergic Targets, β-catenin, and CB1

#### 3.2.1. htr1a (5HT1a Gene)

We found that the UCMS-induced decrease in the 5HT1a gene in the vmPFC was reversed by CBD. This corroborates with the expectation for lower levels of the 5HT1a gene in regions where miR-135 is high, and vice versa [27].

Importantly, we found that the antidepressant-like effects of CBD were mediated by the 5HT1a receptor, as co-administration of CBD and the 5HT1a antagonist, blocked the therapeutic-like effects of CBD in the FST in UCMS rats. This corroborates with a previous study in which CBD was microinjected into the vmPFC in rats exposed to the FST and the OFT [13].

In the NAc, both UCMS and CBD led to a decrease in the 5HT1a gene, as control rats that were treated with vehicle had higher levels of this gene than all the other groups. In the raphe, UCMS or CBD had no effect on 5HT1a gene expression. 

#### 3.2.2. slc6a4 (SERT Gene)

SERT modulation is the main mechanism on which SSRIs are based [56]. Total SERT knockout results in depressive and stressed behavior [57], but variations in its expression in different brain regions can lead to a more complex effect. Therefore, lower expression of the SERT gene was expected to be observed in UCMS rats; we found a decrease in slc6a4 in the PFC, with no effect of CBD treatment. In the NAc, no differences were observed between the groups; in the raphe nucleus, UCMS-vehicle rats demonstrated increased levels compared to controls who were treated with CBD.

As a target of miR-16, levels of the SERT gene were expected to be lower in regions where miR-16 is overexpressed, and vice versa [31,58]. We found that UCMS decreased SERT gene expression in the vmPFC while elevating miR-16. However, even though CBD reversed the effect of UCMS on miR-16 expression in the vmPFC, it did not affect the SERT gene, suggesting other mechanisms which are involved in the regulation of this gene (e.g., miR-15a) [58].

#### 3.2.3. ctnnb1 (β-catenin Gene)

We found that UCMS decreased β-catenin gene levels in the vmPFC and NAc, with no effect in the raphe. This is in line with other studies showing decreased expression of β-catenin in the brain and specifically the PFC and NAc [59,60]. CBD did not restore UCMS-induced downregulation of β-catenin, but in the vmPFC, UCMS rats treated with CBD were not different from any of the other groups. Hence, β-catenin is altered following UCMS exposure, and it regulates microRNA expression [18], but our findings did not demonstrate a robust effect of CBD on β-catenin mRNA in UCMS rats.

In general, increased β-catenin expression correlates with resilience to stress and depression [18,61], and CBD was shown to target the Wnt/β-catenin pathway [62]. 

#### 3.2.4. cnr1 (CB1 Gene)

UCMS decreased the expression of the CB1 gene in the vmPFC, with no effect in the NAc or raphe. In general, CB1 signaling regulates stress responses by modulating the fast feedback inhibition of the hypothalamic–pituitary–adrenal (HPA) axis and its adaptation during exposure to repeated stress [17,63,64,65,66,67,68,69].

CBD functions as a negative allosteric modulator of CB1, and it has been shown to prevent CB1 internalization [70,71]. In our study, CBD did not restore the UCMS-induced decrease in cnr1 in the vmPFC.

## 4. Materials and Methods

### 4.1. Subjects

Male Sprague Dawley rats (60 days old) were group-housed at 22 ± 2 °C under 12 h light/dark cycles (lights turned on at 07:00). Rats were allowed water and laboratory rodent chow ad lib, except when the UCMS procedure required deprivation. The experiments were approved by the University of Haifa Ethics and Animal Care Committee, and adequate measures were taken to minimize pain and discomfort (696/20). 

### 4.2. UCMS Protocol

Rats were subjected to a random sequence of mild stressors [72,73] for 6 weeks. These included cage soiling with 300 mL water, group housing, water and/or food deprivation, reversal of light/dark cycle, cage tilting to 45°, and physical restraint (see supplementary information (SI); Table S1). No UCMS rats were handled and were not subjected to the stress protocol.

### 4.3. Pharmacological Agents

No UCMS and UCMS exposed rats received daily injections (i.p.) of vehicle, CBD (10 mg/kg), or the 5-HT1a-antagonist WAY100635 (WAY; 0.1 mg/kg; Sigma, St. Louis, MO, USA) during the last 3 weeks of the 6-week UCMS model. Drugs were freshly prepared and administered in 1 mL/kg of vehicle. Rats were injected between 15:00 pm and 17:00 pm, irrespective of the stress schedule. Drugs were dissolved in 2% Tween-80 and 98% saline. Doses were based on previous work [74,75].

### 4.4. Behavioral Tests

All tests occurred in a dim light (15–20 lx) and took place between 1300 and 1600 h.

#### 4.4.1. Locomotor Activity and Anxiety-like Behavior

Locomotion was measured in an open-field test (OFT). The open field arena is an open black box 50 × 50 cm in size. It was thoroughly cleaned between each trial. The movements of the rat were recorded and analyzed using Ethovision (version 11) to measure motor activity (over a period of 30 min.) and anxiety-like behavior measured as time spent in the center (first 5 min).

#### 4.4.2. Forced Swim Test (FST)

Conducted in a cylindrical water container (62 cm diameter, 40 cm height, filled with water at a temperature of 22 °C). The water level was such that the rat could only touch the bottom with the tip of its tail. Rats were exposed to the swim tank for 15 min habituation on the first day and 5 min on the second day. Video films of the second day of each FST session were analyzed for passive coping (immobility). An immobility index was calculated: time spent immobile divided by the total time spent in the arena.

### 4.5. Quantitative Real-Time PCR (qRT-PCR)

Rats were sacrificed and brain tissues of the vmPFC, NAc, and raphe nucleus were harvested for molecular analysis (see SI, Figure S1). RNA extraction, cDNA preparation, and qRT-PCR were performed as previously described [76] to detect the expression of miRNAs (miR-16, miR-135, miR-124) and mRNAs (htr1a, slc6a4, ctnnb1 and cnr1; genes coding to 5HT1a, SERT, β-catenin, and CB1r, respectively). For miRNA, 500ng of total RNA was reverse transcribed cDNA using qScript microRNA cDNA Synthesis Kit (Quanta Biosciences, Gaithersburg, MD, USA). For mRNA, 1000 ng of total RNA was converted into cDNA using qScript cDNA Synthesis Kit (Quanta Biosciences, Gaithersburg, MD, USA). This was followed by Real-Time SYBR Green qRT-PCR amplification using specific primers (Quanta Biosciences, Gaithersburg, MD, USA) according to the manufacturer’s instructions. RT reactions were carried out by a Step One real-time PCR system (Applied Biosystems, Waltham, MA, USA). Fold-change values were calculated using the ddCt method relative to the housekeeping gene hypoxanthine phosphoribosyl transferase RNU6 (miRNA) or HPRT (mRNA). Primers for both miRNAs (miR-16-5p, miR-124-5p, and miR-135a-5p) and mRNAs (see Table 5) were designed and synthesized by Agentek (Tel Aviv, Israel). Primer suitability was determined using standard curve analysis, melting curve analysis, and linearity and threshold.

### 4.6. Statistical Analysis

The results are expressed as means ± SEM. For statistical analysis, one-way ANOVA, two-way ANOVA, and Pearson bivariate correlation test were used as indicated. All post hoc comparisons were made using Tukey’s range test. Significance was set at *p* ≤ 0.05. Data were analyzed using SPSS 27 (IBM, Chicago, IL, USA). Normality assumption was examined using the Kolmogorov–Smirnov and Shapiro–Wilk tests.

## 5. Conclusions

We show for the first time that CBD can prevent UCMS-induced increases in vmPFC miR-16 and miR-135. The antidepressant effects of CBD in rats exposed to the UCMS model for depression were mediated by the 5HT1a receptor. 

CBD seems to have positive effects of diminishing depressive-like behaviors with the advantage of not being addictive or having many side effects [77]. However, the mechanisms underlying its therapeutic effects are still not entirely clear and involve multiple targets.

## Figures and Tables

**Table 1:** Pearson correlation coefficients between miRNA levels and behavioral measures in rats exposed to UCMS and CBD.

	miR-16 vmPFC	miR-16 NAC	miR-16 Raphe	miR-124 vmPFC	miR-124 NAC	miR-124 Raphe	miR-135 vmPFC	miR-135 NAC	miR-135 Raphe
FST—immobility	r = 0.277 *p* = 0.102	r = 0.250 *p* = 0.147	r = 0.069 *p* = 716	r = 0.087 *p* = 0.613	r = −0.040 *p* = 0.816	r = 0.054 *p* = 0.749	r = 0.428 * ***p* = 0.012**	r = −0.339 * ***p* = 0.046**	r = 0.187 *p* = 0.340
OFT—total distance	r = 0.176 *p* = 0.305	r = 0.322 *p* = 0.060	r = −0.473 ** ***p* = 0.008**	r = −0.159 *p* = 0.355	r = 0.455 ** ***p* = 0.005**	r = 0.060 *p* = 0.718	r = 0.012 *p* = 0.944	r = 0.192 *p* = 0.268	r = 0.474 * ***p* = 0.011**
OFT—time in center	r = 0.0.14 *p* = 0.933	r = −0.2.33 *p* = 0.198	r = 0.276 *p* = *0*.140	r = −0.184 *p* = 0.282	r = −0.142 *p* = 0.403	r = 0.157 *p* = 0.347	r = −0.75 *p* = 0.675	r = −0.229 *p* = 0.186	r = −0.70 *p* = 0.723

FST—forced swim test; OFT—open field test; vmPFC: ventromedial prefrontal cortex; NAc: nucleus accumbens; miR: microRNA; UCMS: unpredictable chronic mild stress; CBD: cannabidiol. *, *p* < 0.05, **, *p* < 0.01.

**Table 2:** Pearson correlation coefficients between miRNA levels and genes in the vmPFC in rats exposed to UCMS and CBD.

	hrt1a	slc6a4	ctnnb1	cnr1
**miR-16**	r = 0.591 *** ***p* = 0.000**	r = −0.432 * ***p* = 0.031**	r = 0.140 *p* = 0.436	r = 0.250 *p* = 0.160
**miR-124**	r = −0.131 *p* = 0.469	r = 0.281 *p* = 0.183	r = 0.027 *p* = 0.880	r = −0.075 *p =* 0.682
**miR-135**	r = 0.478 ** ***p* = 0.008**	r = −0.346 *p* = 0.091	r = 0.001 *p* = 0.997	r = 0.258 *p* = 0.154

*, *p* < 0.05, **, *p* < 0.01, ***, *p* < 0.001.

**Table 3:** Pearson correlation coefficients between miRNA levels and genes in the NAc in rats exposed to UCMS and CBD.

	hrt1a	slc6a4	ctnnb1	cnr1
**miR-16**	r = −0.404 *p* = 0.051	r = −0.33 *p* = 0.878	r = 0.479 ** ***p* = 0.006**	r = 0.229 *p* = 0.215
**miR-124**	r = −0.272 *p* = 0.198	r = −0.56 *p* = 0.794	r = 0.463 ** ***p* = 0.008**	r = 0.224 *p* = 0.234
**miR-135**	r = −0.324 *p* = 0.114	r = −0.080 *p* = 0.724	r = 0.222 *p* = *0*.222	r = 0.226 *p* = 0.231

**, *p* < 0.01.

**Table 4:** Pearson correlation coefficients between miRNA levels and genes in the raphe nucleus in rats exposed to UCMS and CBD.

	hrt1a	slc6a4	ctnnb1	cnr1
**miR-16**	r = 0.125 *p* = 0.579	r = 0.064 *p* = 0.795	r = −0.205 *p* = 0.325	r = 0.012 *p* = 0.956
**miR-124**	r = −0.358 *p* = 0.056	r = 0.408 * ***p* = 0.035**	r = −0.175 *p* = 0.330	r = 0.147 *p* = 0.438
**miR-135**	r = 0.191 *p* = 0.382	r = 0.232 *p* = 0.298	r = 0.050 *p* = 0.815	r = 0.324 *p* = 0.142

*, *p* < 0.05.

**Table 5:** Primers for mRNAs used for real-time PCR.

Name	Description	GeneBankID (NM)	Protein Name	Primer Sequence	Efficacy	Description
*Hprt*	Housekeeping gene; used as a reference gene	NM_012583.2	HPRT	F: 5′CGCCAGCTTCCTCCTCAG3′	NM_012583.2	HPRT
*Htr1a*	Serotonergic auto-receptor	R: 5′ATAACCTGGTTCATCATCACTAATCAC3′	99.83		R: 5′ATAACCTGGTTCATCATCACTAATCAC3′	99.83
*Slc6a4*	The serotonergic transporter	NM_012585.1	5HT1a	F: 5′CCACGGCTACACCATCTACTC3′	NM_012585.1	5HT1a

F: forward primer; R: reverse primer.

## Data Availability

Not applicable.

## Supplementary Materials

The following supporting information can be downloaded at: https://www.mdpi.com/article/10.3390/ijms24032052/s1.
