# Supplementary material for: Cannabidiol Modulates Alterations in PFC microRNAs in a Rat Model of Depression
Source: Int J Mol Sci. 2023 Jan 20;24(3):2052. doi: 10.3390/ijms24032052 (PMC9953518; doi:10.3390/ijms24032052)
Supplement: Supplementary file 1 [file ijms-24-02052-s001.zip › ijms-2144745-supplementary.pdf]

Supplementary information

Table S1: UCMS schedule

			S	M	T	W	T	F	S
Week 1	Food deprivation		13 ▶	9		13 ▶	9		
	Water deprivation				16 ▶	10		16 ▶	10
	Empty water bottle					10-11			
	Cage tilt 45°			16 ▶	9			16 ▶	9
	Overnight illumination		18 ▶	7			18 ▶	7	
	Soiled cage						12 ▶	9	
	Physical restraint		10-12					10-12	
	Pair-housing					10 ▶	10		10 ▶
			S	M	T	W	T	F	S
Week 2	Food deprivation		13 ▶	9		13 ▶	9		
	Water deprivation			16 ▶	10		16 ▶	10	
	Empty water bottle				10-11				
	Cage tilt 45°		16 ▶	9		16 ▶	9		
	Overnight illumination			18 ▶	7		18 ▶	7	
	Soiled cage				12 ▶	9			
	Physical restraint			10-12		10-12			
	Pair-housing	▶	10		10 ▶	10			
			S	M	T	W	T	F	S
Week 3	Food deprivation			13 ▶	9		13 ▶	9	
	Water deprivation		16 ▶	10		16 ▶	10		
	Empty water bottle						10-11		
	Cage tilt 45°		16 ▶	9			16 ▶	9	
	Overnight illumination			18 ▶	7	18 ▶	7		
	Soiled cage				12 ▶	9			
	Physical restraint		10-12			10-12			
	Pair-housing							10 ▶	10
			S	M	T	W	T	F	S
Week 4	Food deprivation		13 ▶	9		13 ▶	9		
	Water deprivation				16 ▶	10		16 ▶	10
	Empty water bottle					10-11			
	Cage tilt 45°			16 ▶	9			16 ▶	9
	Overnight illumination		18 ▶	7			18 ▶	7	
	Soiled cage						12 ▶	9	
	Physical restraint		10-12					10-12	
	Pair-housing					10 ▶	10		10 ▶
			S	M	T	W	T	F	S
Week 5	Food deprivation		13 ▶	9		13 ▶	9		
	Water deprivation			16 ▶	10		16 ▶	10	
	Empty water bottle				10-11				
	Cage tilt 45°		16 ▶	9		16 ▶	9		
	Overnight illumination			18 ▶	7		18 ▶	7	
	Soiled cage				12 ▶	9			
	Physical restraint			10-12		10-12			
	Pair-housing	▶	10		10 ▶	10			
			S	M	T	W	T	F	S
Week 6	Food deprivation			13 ▶	9		13 ▶	9	
	Water deprivation		16 ▶	10		16 ▶	10		
	Empty water bottle						10-11		
	Cage tilt 45°		16 ▶	9			16 ▶	9	
	Overnight illumination			18 ▶	7	18 ▶	7		
	Soiled cage				12 ▶	9			
	Physical restraint		10-12			10-12			
	Pair-housing							10 ▶	10

The arrow indicates the duration of the stressor

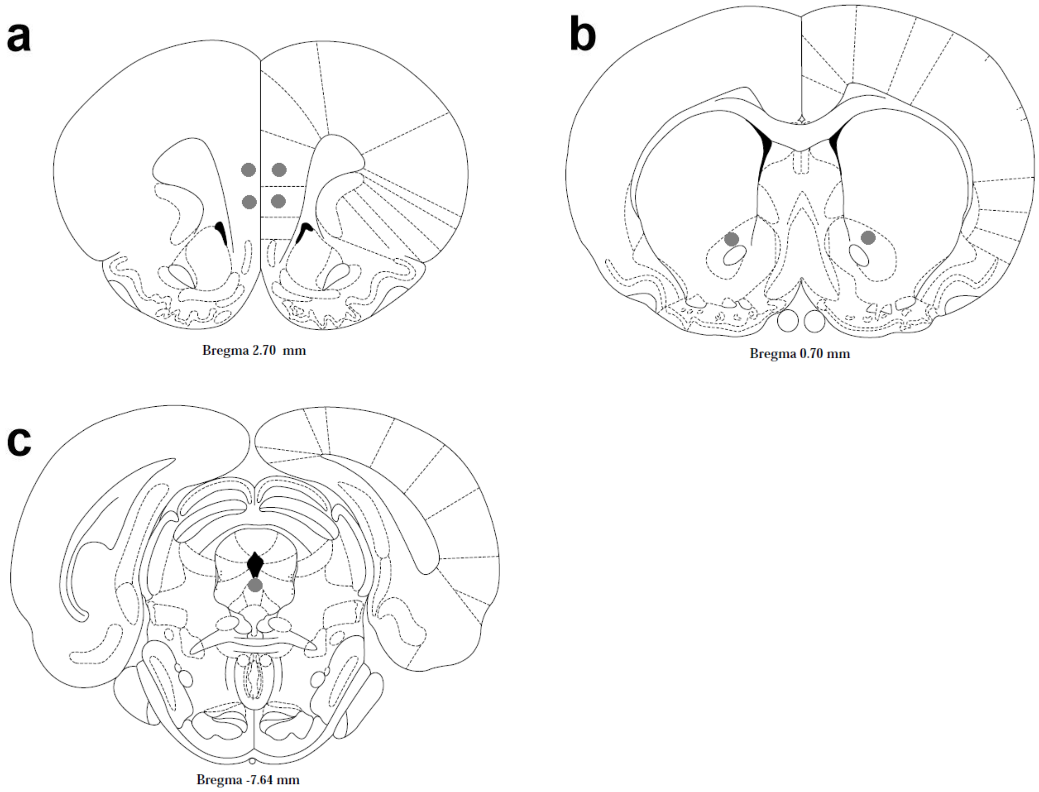

Figure S1: Rat brain atlas illustrations indicating punch locations. The numbers refer to the distance from Bregma. Bilateral punches (1mm diameter) were obtained from the (a) vmPFC (b) NAc and (c) raphe nucleus.
